# Intramuscular Ketamine Effect on Postnasal Surgery Agitation: A Prospective Double-Blinded Randomized Controlled Trial

**DOI:** 10.1155/2023/2286451

**Published:** 2023-02-27

**Authors:** Husam A. Almajali, Ali M. Abu Dalo, Nidal M. Al-Soud, Ali Almajali, Abdelrazzaq Alrfooh, Thani Alawamreh, Hamza Al-Wreikat

**Affiliations:** ^1^Department of Anaesthesia and Intensive Care, Jordanian Royal Medical Services, Amman, Jordan; ^2^Department of ENT, Jordanian Royal Medical Services, Amman, Jordan

## Abstract

This study investigates the effect of intramuscular ketamine on emergence agitation (EA) following septoplasty and open septorhinoplasty (OSRP) when administered at subanesthetic doses at the end of surgery. A random sample of 160 ASA I-II adult patients who underwent septoplasty or OSRP between May and October, 2022, was divided into two groups of eighty patients each: ketamine (Group K) and saline (Group S) with the latter serving as the control group. At the end of surgery immediately after turning off the inhalational agent, Group K was administered with intramuscular 2 ml of normal saline containing 0.7 mg/kg ketamine and Group S with 2 ml of intramuscular normal saline. Sedation and agitation scores at emergence from anesthesia were recorded after extubation using the Richmond Agitation-Sedation Scale (RASS). The incidence of EA was higher in the saline group than in the ketamine group (56.3% vs. 5%; odds ratio (OR): 0.033; 95% confidence interval (CI): 0.010–0.103; *p* < 0.001). Variables associated with a higher incidence of agitation were ASA II classification (OR: 3.286; 95% (CI): 1.359–7.944; *p*=0.008), longer duration of surgery (OR: 1.010; 95% CI: 1.001–1.020; *p*=0.031), and OSRP surgery (OR: 2.157; CI: 1.056–5.999; *p*=0.037). The study concluded that the administration of intramuscular ketamine at a dose of 0.7 mg/kg at the end of surgery effectively reduced the incidence of EA in septoplasty and OSRP surgery.

## 1. Introduction

Dealing with an agitated patient can be challenging. Emergence agitation (henceforth, EA) is a postanesthetic phenomenon characterized by restlessness, excitation, and confusion during early recovery from general anesthesia [1] in around 22.2% of patients[2]. The precise cause of EA is still unknown, since one retrospective cohort study attributed EA to the sense of suffocation caused by nasal packing following surgeries [3], and another claimed the opposite, i.e., nasal packing did not increase the incidence of EA [4]. Other potential risk factors include ASA (American Society of Anesthesiologists) physical status ≥2, young age, postoperative pain, male gender, inhalational anesthesia, postoperative nausea and vomiting (PONV), oral cavity and ENT surgeries, current smoking, and the presence of a tracheal tube [2, 4].

Although EA spontaneously resolves typically after a short period [5], it may lead to serious consequences such as bleeding, self-extubation, prolonged hospital admission, and injury to the patient and theatre staff [6, 7]. Thus, eliminating risk factors and adequate postoperative pain control leading to the effective prevention of EA will improve clinical outcomes [2].

Studies have investigated different drugs and found some to be effective in EA prevention including opioids, alpha 2 receptor agonists such as dexmedetomidine, and ketamine [3, 9, 10]. Ketamine, an N-methyl-D-aspartate (NMDA) receptor antagonist used as an anesthetic agent, has an analgesic effect at subanesthetic doses [10]. The peak plasma concentrations of ketamine following IV and IM administration occur within 1 to 5 minutes, respectively [11]. To the best of the researchers' knowledge, the effect of ketamine on EA following nasal surgeries has been investigated only when it was intravenously administered.

This study investigates the effect of ketamine on EA following septoplasty and open septorhinoplasty (henceforth, OSRP) when intramuscularly administered at subanesthetic doses to reduce the incidence of EA. The researchers resorted to intramuscular ketamine injection to reduce the incidence of EA without causing significant sedation and without increasing the response time to verbal stimulus, as drug absorption and the onset of action will be slower than the IV route investigated earlier [9]. The primary outcome of the study was developing agitation (RASS ≥ 2), and the secondary is the numerical rating scale of pain (NRS) ≥5.

## 2. Materials and Methods

**Figure 1:**
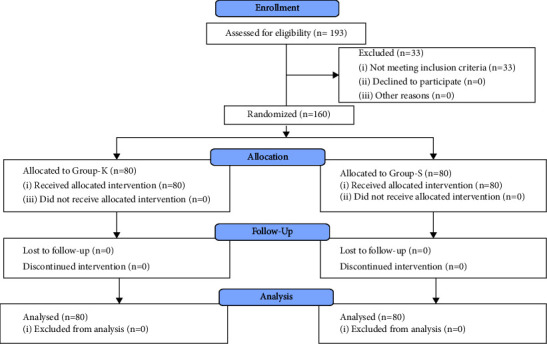
Consort flow diagram.

This multicenter randomized study, the steps of which are given in Figure 1, was performed between May and October, 2022, after it was approved by the Royal Medical Services Human Research Ethics Committee (registration number NCT05313659 at https://clinicaltrials.gov). The sample included patients aged 18–64 with ASA physical status I-II scheduled for septoplasty and OSRP from whom a written consent was obtained. Patients with ketamine or morphine allergies, a history of cardiac, neurological, or psychiatric disease, glaucoma, and a body mass index of less than 20 or more than 30 kg/m^2^ were excluded.

To achieve the purpose of the study using the GPower computer program, considering alpha set at 0.05 with medium effect size, a study power of 80%, and a sample size of 158 and 79 in each group was deemed suitable. Thus, the researchers opted for a sample of 160 patients, from the 193 patients scheduled for septoplasty or open septorhinoplasty who were first assessed for eligibility which resulted in the exclusion of 33 patients: 17 with a BMI <20 or >30, 8 under 18 years of age, 4 with a history of hypertension, 3 with a psychiatric problem, and 1 with morphine allergy. The 160 patients were randomly distributed into two equal groups: the ketamine group (Group K) and the saline (Group S) group. The demographical and clinical data are summarized in Table 1.

Random Allocation software was used to provide simple random codes for ketamine and normal saline syringes, all of which were prepared and sealed in sequentially coded identical opaque containers as per the allocation sequence of the specialist physician who partook in the randomization process and was excluded from the follow-up. The anesthetic nurse provided the sequential opaque container just before turning off the inhalational agent, and the researchers documented the syringe code. Patients, researchers, outcome assessors, and anesthetic nurses were all kept blinded for allocation.

### 2.1. General Anesthesia and Surgical Procedure

The patients were taken to the operating room without any premedication. Standard AAGBI monitors were used. Anesthesia was intravenously induced by 1.5 *µ*g/kg fentanyl, 2 mg/kg propofol, and 0.6 mg/kg rocuronium which was maintained through a mixture of 50% oxygen and 1.2% isoflurane in the air at a flow rate of 2.5 L/min. For intraoperative analgesia and hypotensive technique, remifentanil infusion at a rate of 0.05–2 *µ*g/kg/min was used. Following intubation, all patients received 8 mg of dexamethasone as prophylaxis for postoperative nausea and vomiting (henceforth, PONV). The ventilator parameters were adjusted at a tidal volume of 7–10 ml/kg and a respiratory rate of 10–12 breaths/min to maintain the end-tidal CO2 levels of 30–35 mmHg. Remifentanil infusion was stopped 10 minutes before the end of the surgery to prevent any delay in the emergence from anesthesia. At the end of the surgery immediately after the inhalational agent was discontinued, 2 ml of normal saline containing 0.7 mg/kg of racemic ketamine was intramuscularly administered to Group K, and only 2 ml of normal saline was administered to Group S using a 3 ml syringe. The injection site of both groups was the lateral thigh. For postoperative analgesia, 0.07 mg/kg of intravenous morphine was also administered when the inhalational agent was turned off, and a nasal pack was used for all patients. The patients were ventilated with 100% oxygen at a flow rate of 7 l/min and then extubated once they met extubation criteria.

The patients' EA level was evaluated using the Richmond Agitation-Sedation Scale (RASS) immediately after extubation until they were handed over to the postanesthaesia care unit (henceforth, PACU), and the highest score documented, is shown in Table 2 (adapted from [12]). For the purpose of this study, patients with a RASS score of +2 or more were considered to have EA.

During the first 30 minutes in the PACU, the pain score was evaluated using the numerical rating scale (NRS) of 0–10 in which 0 equaled no pain and 10 was the worst possible pain. Any patient reporting a pain score of 5 or more was given 1 g of intravenous paracetamol. Any patient experiencing PONV was given 4 mg of ondansetron.

### 2.2. Statistics

The categorical data were presented in frequency and percentages, and continuous variables were presented as mean ± standard deviation. The chi-square of independence was used to explore the bivariate association between the categorical data. To compare continuous variables, the independent *t* test was used alongside the multivariable analysis by the backward binary stepwise logistic regression model to predict the risk factors for postoperative agitation; any variables significant at *p* ≤ 0.20 in the bivariate analysis were nominated for entry into the regression model and were reported as the odds ratio (OR) with 95% confidence interval (CI). The Hosmer–Lemeshow test was used to express model goodness-of-fit.

A *p* value of <0.05 was considered statistically significant. Statistical analyses were performed using the SPSS for Windows (version 28; IBM Corporation).

## 3. Results

As given in Table 3, the bivariate analysis shows that the incidence of EA in the saline group was 56.3% but only 5% in the ketamine group (*p* ≤ 0.001).

The other variables with significant differences between agitated and nonagitated patients were ASA physical status classification (*p*=0.020), age (*p*=0.034), pain score (*p*=0.006), and duration of surgery (*p*=0.029). In addition to these five variables, an extra variable associated with EA (*p* ≤ 0.2) in the bivariate analysis (the type of surgery) was entered into the multivariate backward binary logistic regression model, and the following four variables were found to be significantly associated with EA: study arms, ASA physical status classification, duration of surgery, and type of surgery, as shown in Table 4. The EA odds were 0.033 times lower in Group K than in Group S which means that the incidence of EA has been reduced by 96.7% in Group K.

On the other hand, the EA odds were 3.3 times higher in ASA II than in ASA I, and 2.2 times higher in OSRP than in septoplasty surgery. They were also expected to increase by 1.01 times per minute for the duration of the surgery.

Table 5 shows the time taken to obtain a response to verbal stimuli after extubation, the incidence of postoperative pain, PONV, and additional antiemetic requirements in the PACU. While the time taken to obtain a verbal response to verbal stimuli was higher in Group K (*p* < 0.002) with a mean difference of 0.48 minutes (29 seconds), but the postoperative pain score was significantly lower in this group than that of Group S (*p* < 0.001). There were no statistically significant differences between the study arms when considering PONV occurrence and additional antiemetic requirements in the PACU.

## 4. Discussion

Intramuscularly administering a reduced dose of ketamine at the end of surgery was effective in reducing the incidence of EA in adults who underwent septoplasty or OSRP. However, this accompanied a 29-second delay in response to verbal stimuli. The pain scores (NRS) were lower for Group K. The risk factors for agitation included ASA II physical status, longer surgical duration, and OSRP surgery. No clinically significant differences were noted between study groups in terms of gender, type of surgery, ASA classification, age, and BMI (a *p* value >0.05 for all variables).

The incidence of EA was 56.3%, which is higher than or almost similar to that reported by some earlier studies [2, 4] that investigated the incidence of EA following nasal surgery. This difference may be attributed to the type of nasal surgery, certain risk factors, or the absence of a specific scale for evaluating postoperative agitation in each study. The efficacy of ketamine in EA prevention after nasal surgery has been investigated, and Abitağaoğlu et al. [13] reported that the intravenous administration of ketamine after anesthesia induction did not affect the incidence of EA in adults who underwent septoplasty surgeries and delayed the response to verbal stimuli. Demir and Yuzkat [9] reported that the effect of ketamine intravenously administered 20 minutes before the end of surgery on 140 patients who underwent rhinoplasty showed that ketamine reduced the incidence of EA at subanesthetic doses but prolonged the duration of anesthesia.

In a study on the effect of intramuscular ketamine as a sedative agent on severely agitated patients in the emergency department, the results were inconclusive as to its dose that may produce dissociation, which was already established as 3-4 mg/kg [14]. Intramuscular ketamine at a dose of <5 mg/kg provides adequate sedation with a low risk for intubation [15], but a dose of 4 mg/kg was effective in producing sedation for severe agitation but with an increased risk of intubation [16]. Reducing the dose to 2 mg/kg, O'Brien et al. [17] reported that intramuscular ketamine caused adequate sedation and was effective for severe agitation without the need for intubation. In this study, the smaller 0.7 mg/kg dose significantly reduced the incidence of agitation following septoplasty and OSRP, and caused light sedation (RASS = −2) in 26 patients out of the 80 patients of Group K.

Isoflurane was used to maintain anesthesia because the literature has related that there is an increased incidence of EA to agents with low blood-gas partition coefficients such as sevoflurane and desflurane [4, 18–20]. Pain has been considered an important independent risk factor for EA [4, 21]. Higher doses of intraoperative opioids were associated with a higher incidence of agitation [20], which is why the researchers administered a lower dose of morphine. In contrast to previous research, this study shows that pain is significantly associated with agitation in bivariate analysis but not a significant predictor of EA in multivariant binary logistic regression.

Research on the EA risk factors after nasal surgery listed the following factors as risk factors: youth, male gender, postoperative pain, inhalational anesthesia, smoking, PONV, and the presence of a tracheal tube and a urinary catheter. [4, 2], [22], [23] Although the male gender was considered a risk factor in previous studies, there was no significant difference in the incidence of EA between the 85 males and 75 females in this study. The ASA II physical status proved to be a significant risk factor for EA which could be attributed to the fact that many patients in this study were smokers. Both the duration and type of nasal surgery were found to be significant risk factors which disprove previous research findings [4]. As the study was multicenter, the surgical procedures were performed by different surgeons which may contribute to the variation in the duration of the surgery and can be considered a limitation.

## 5. Conclusion

Intramuscular ketamine is highly effective in preventing EA following septoplasty and OSRP when administered at a dose of 0.7 mg/kg at the end of surgery. Although it is difficult to prevent EA completely, it can be reduced by modifying risk factors whenever possible. While intramuscular ketamine was preventive, ASA II physical status, longer duration of surgery, and OSRP surgery were the main risk factors for EA development.

## Figures and Tables

**Table 1:** Demographical and clinical data of the sample.

Variables	Category	*N* (%)	*Study's arms (N* *=* *160)*	
			Saline *n* (%)	Ketamine *n* (%)
Gender	Male Female	85 (53.1) 75 (46.9)	43 (53.8) 37 (46.3)	42 (52.5) 38 (47.5)

Surgery	Septoplasty OSRP	82 (51.2) 78 (48.8)	41 (51.2) 39 (48.8)	41 (51.2) 39 (48.8)

ASA	I II	71 (44.4) 89 (55.6)	35 (43.8) 45 (56.3)	36 (45.0) 44 (55.0)

Age (years)	Mean ± SD	28 ± 7.9	27.2 ± 7.5	28.8 ± 8

BMI	Mean ± SD	24 ± 2.9	24.2 ± 2.9	23.9 ± 2.9

OSRP = open septorhinoplasty; ASA = American Society of Anesthesiologists physical status classification; BMI = body mass index.

**Table 2:** Richmond Agitation-Sedation Scale.

Score	Term	Description
+4	Combative	Overtly combative or violent; immediate danger to staff
+3	Very agitated	Pulls on or removes tube(s) or catheter(s) or has aggressive behavior towards staff
+2	Agitated	Frequent nonpurposeful movement or patient-ventilator dyssynchrony
+1	Restless	Anxious or apprehensive but movements not aggressive or vigorous
0	Alert and calm	
−1	Drowsy	Not fully alert, but has sustained (more than 10 seconds) awakening, with eye contact, to voice
−2	Light sedation	Briefly (less than 10 seconds) awakens with eye contact to voice
−3	Moderate sedation	Any movement (but no eye contact) to voice
−4	Deep sedation	No response to voice, but any movement to physical stimulation
−5	Unarousable	No response to voice or physical stimulation

**Table 3:** Bivariate analysis for risk factors of postoperative agitation.

Variables	Category	*Agitation*		Test value	*p* value
		Nonagitated *n* (%)	Agitated *n* (%)		
Study's arms	Saline Ketamine	35 (43.8) 76 (95.0)	45 (56.3) 4 (5.0)	49.45	**≤0.001** ^**a**^

Gender	Male Female	56 (65.9) 55 (73.3)	29 (34.1) 20 (26.7)	1.041	0.308^a^

Type of surgery	Septoplasty OSRP	62 (75.6) 49 (62.8)	20 (24.4) 29 (37.2)	3.078	0.079^a^

ASA	I II	56 (78.9) 55 (61.8)	15 (21.1) 34 (38.2)	5.420	**0.020** ^**a**^

Age (years)	Mean ± SD	28.92 ± 8.52	26.02 ± 6.18	2.145	0.034^b^

BMI	Mean ± SD	24.01 ± 2.98	24.12 ± 2.89	0.223	0.824^b^

Pain score	Mean ± SD	3.44 ± 1.73	4.24 ± 1.56	2.793	**0.006** ^**b**^

Duration of Surgery (min)	Mean ± SD	107.03 ± 46.18	124 ± 49.37	2.209	**0.029** ^**b**^

a = chi-square test; b = independent *t*-test; SD = standard deviation. These are the P values of the factors that found to have significant association with EA in the bivariate analysis.

**Table 4:** Multivariate binary logistic regression analysis to predict agitation.

Variables	*B*	S.E	Wald	Adjusted OR (95% CI)	*p* value
*Study's arms*					**≤0.001**
Ketamine	−3.425	0.589	33.860	0.033 (0.010–0.103)	
Saline				1.0 (reference)	
*ASA*					**0.008**
I	1.190	0.450	6.980	3.286 (1.359–7.944)	
II				1.0 (reference)	
Duration of surgery	0.010	0.005	4.664	1.010 (1.001–1.020)	**0.031**
*Type of surgery*					**0.037**
OSRP	0.923	0.443	4.335	2.157 (1.056–5.999)	
Septoplasty				1.0 (reference)	

Nagelkerke *R* square = 0.457,; Hosmer Lemesho test (*X*^2^ = 5.411, *p*=0.713).

**Table 5:** Postoperative follow-up data.

Variables		*Groups*		Test value	*p* value
		Normal saline	Ketamine		
PONV	No	68 (85.0%)	61 (76.3%)	1.960^a^	0.161
	Yes	12 (15.0%)	19 (23.8%)		

Additional antiemetic requirement	No	78 (97.5%)	74 (92.5%)	2.105	0.147
	Yes	2 (2.5%)	6 (7.5%)		

Time to verbal response (min)		2.90 ± 1.03	3.38 ± 0.92	3.086^b^	**0.002**

Postoperative pain		4.61 ± 1.53	2.76 ± 1.35	8.102^b^	**<0.001**

a = chi-square test; b = independent *t*-test; PONV = postoperative nausea and vomiting. These are the P values of the factors that found to have clinically significant difference in the results between the two arms of the study.

## Data Availability

The data used to support the findings of this study are included within the article.
